# Cerebellar liponeurocytoma – a rare entity: a case report

**DOI:** 10.1186/s13256-018-1706-z

**Published:** 2018-06-17

**Authors:** Oliver Gembruch, Andreas Junker, Yahya Ahmadipour, Ulrich Sure, Elias Lemonas

**Affiliations:** 1 Department of Neurosurgery, University Hospital Essen, University of Duisburg-Essen, Hufelandstrasse 55, 45122 Essen, Germany; 2 Department of Neuropathology, University Hospital Essen, University of Duisburg-Essen, Essen, Germany

**Keywords:** Cerebellar liponeurocytoma, Cerebellar neoplasm, Radiotherapy

## Abstract

**Background:**

Cerebellar liponeurocytoma is a rare tumor of the central nervous system occurring mainly in the posterior fossa, which shows neuronal and variable astrocytic differentiation with foci of lipomatous differentiation. Liponeurocytoma develops in adult patients and is defined in the World Health Organization classification of 2016 as a rare benign grade II tumor.

**Case presentation:**

A 39-year-old Italian man presented to our department suffering from headache and nausea. Magnetic resonance imaging revealed a right-sided cerebellar lesion showing poor contrast enhancement without an obstructive hydrocephalus. Surgery was indicated and total tumor resection was achieved. He was discharged without any neurological deficits. Histopathological examinations revealed a cerebellar liponeurocytoma. A neurological follow-up examination revealed no neurological deficit directly after surgery and 1 year later. Radiotherapy was recommended at the neurooncological board despite the total removal of the tumor, but our patient refused adjuvant radiotherapy. Magnetic resonance imaging of his neurocranium with and without contrast enhancement 48 hours after surgery and 15 months after surgery showed no residual tumor.

**Conclusions:**

Liponeurocytomas are rare benign tumors occurring in the majority of cases in the cerebellum. The therapy of choice is surgery. Postoperative radiotherapy has to be discussed individually, but seems to be sufficient if complete tumor resection is not achieved or in cases of a tumor recurrence.

## Background

Cerebellar liponeurocytoma is a rare tumor entity mainly located in the posterior cranial fossa and typically developing in adults with a favorable clinical prognosis. It is histologically characterized by lipidized cells found in clusters or scattered between small neoplastic cells. Immunohistochemical staining shows advanced neuronal/neurocytic differentiation with a low proliferation index.

Liponeurocytoma was first described in 1978 by Bechtel *et al.* as a mixed mesenchymal and neuroectodermal tumor in a 44-year-old man with a cerebellar lesion [1]. Some previous cases were published as “unusual medulloblastomas in adults,” “lipomatous medulloblastomas,” “lipidized medulloblastomas,” “neurolipocytomas,” or “medullocytomas.”

The World Health Organization (WHO) recognized it in the 2000 WHO classification as a cerebellar liponeurocytoma emphasizing its neurocytic differentiation and classified it in the group of neuronal tumors [2]. In the WHO classification of 2016, the tumor was classified as a glioneuronal tumor and was upgraded to grade II due to the results of long-term follow-up studies showing a higher recurrence rate than originally expected [3].

Although findings from computed tomography (CT) and magnetic resonance imaging (MRI), and pathological and immunohistochemical characteristics have been reported in around 70 cases, clinical and pathological diagnosis of liponeurocytoma remained challenging and the characteristics of this tumor entity are still poorly understood.

Accurate differential diagnosis of this rare central nervous system (CNS) tumor from other brain tumors such as medulloblastoma or oligodendroglioma is important to avoid unnecessary aggressive adjuvant therapies.

This case report presents a rare case of a liponeurocytoma located in the posterior fossa and summarizes the typical radiological and histopathological features of this rare tumor entity. Tumor recurrence, adjuvant therapy, and important differential diagnoses are discussed.

## Case presentation

**Fig. 1:**
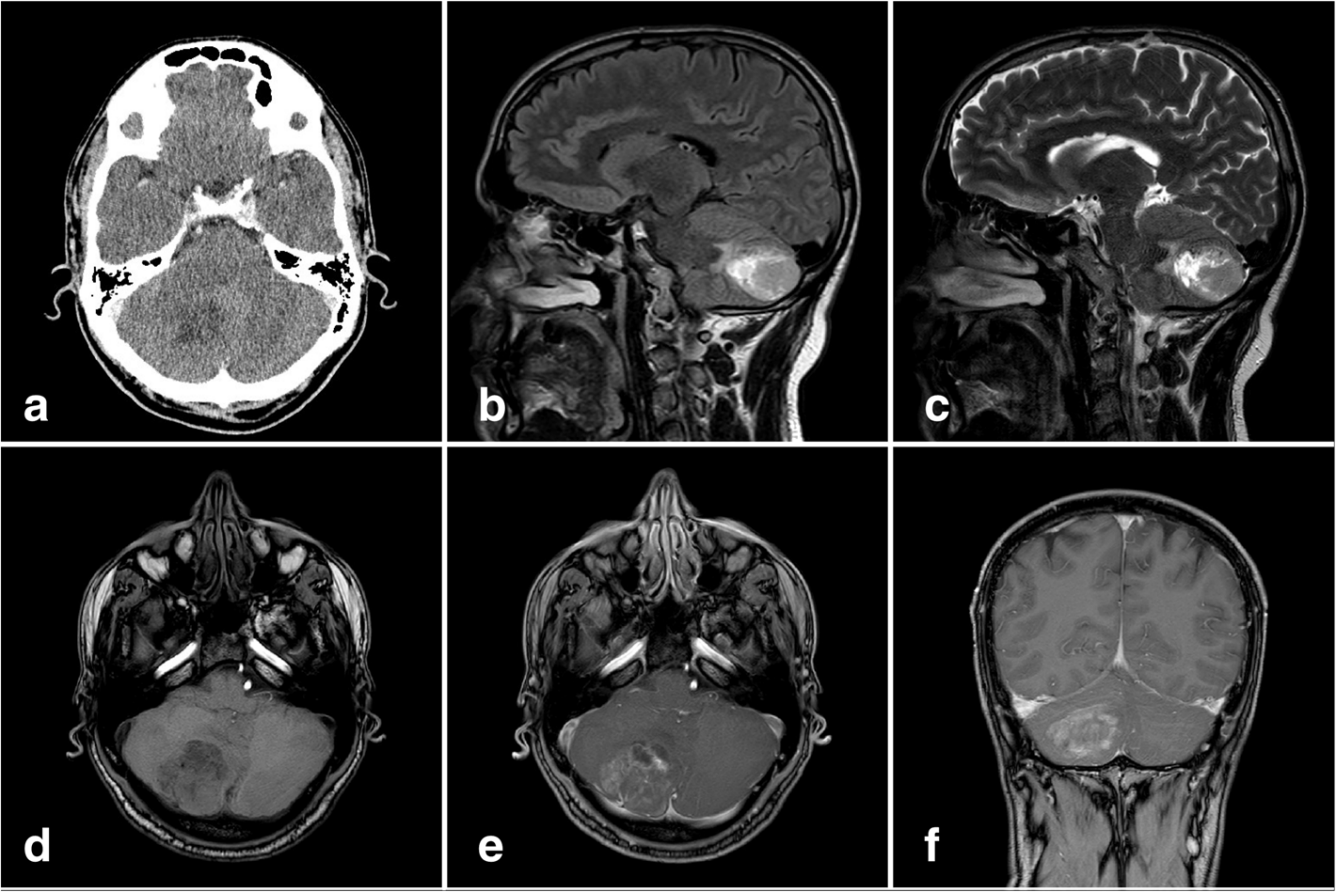
Computed tomography shows a hypointense ill-defined lesion of the right cerebellar hemisphere (**a**). The lesion presents a hyperintensity on fluid-attenuated inversion recovery sequence (**b**) and T2-weighted imaging (**c**). On T1-weighted imaging, the lesion shows a hypointensity (**d**). A light, diffuse contrast enhancement can be observed on T1-weighted imaging with contrast (**e** + **f**)

A 39-year-old Italian man presented to our department suffering from headache and nausea over the past months. CT and MRI revealed an ill-defined, 39 × 37 × 29 mm (anterior-posterior×transverse×cranial-caudal) tumor. On CT, the lesion presented as slightly hypointense with poor contrast enhancement. On MRI, a hyperintensity on fluid-attenuated inversion recovery (FLAIR) sequence and on T2-weighted imaging was detected. On T1-weighted imaging, the lesion showed a hypointensity. The lesion showed poor contrast enhancement of the right cerebellar hemisphere without an obstructive hydrocephalus on T1-weighted images with contrast enhancement (Fig. 1).

Our patient did not suffer from any other comorbidities; he had not undergone any surgeries. He did not use medication. He had never consumed alcohol, smoked tobacco, or used other drugs. He is married, has two children, and works as a cook in a family owned restaurant. Similar cases were not reported in his family; no relatives had suffered from a tumor in the past. Neurological examinations at admission showed no sensorimotor deficits, no cranial nerve deficits, normal response of his reflexes, and normal standing and walking abilities without any unstableness. Blood pressure, pulse, temperature, and laboratory findings (that is, complete blood count, liver function, renal function, and C-reactive protein) were within normal range.

Surgery was indicated and written consent was obtained. Surgery was performed under general anesthesia with our patient in a semi-sitting position. Monitoring was done with somatosensory and muscle-evoked potentials. A right-sided suboccipital craniotomy was performed. On intraoperative examination, we observed a glassy gray-black tumor that was not well demarcated from the surrounding tissue. Piecemeal tumor removal was performed by microsurgical technique using the Sonoca 300 (Söring GmBH, Quickborn, Germany).

**Fig. 2:**
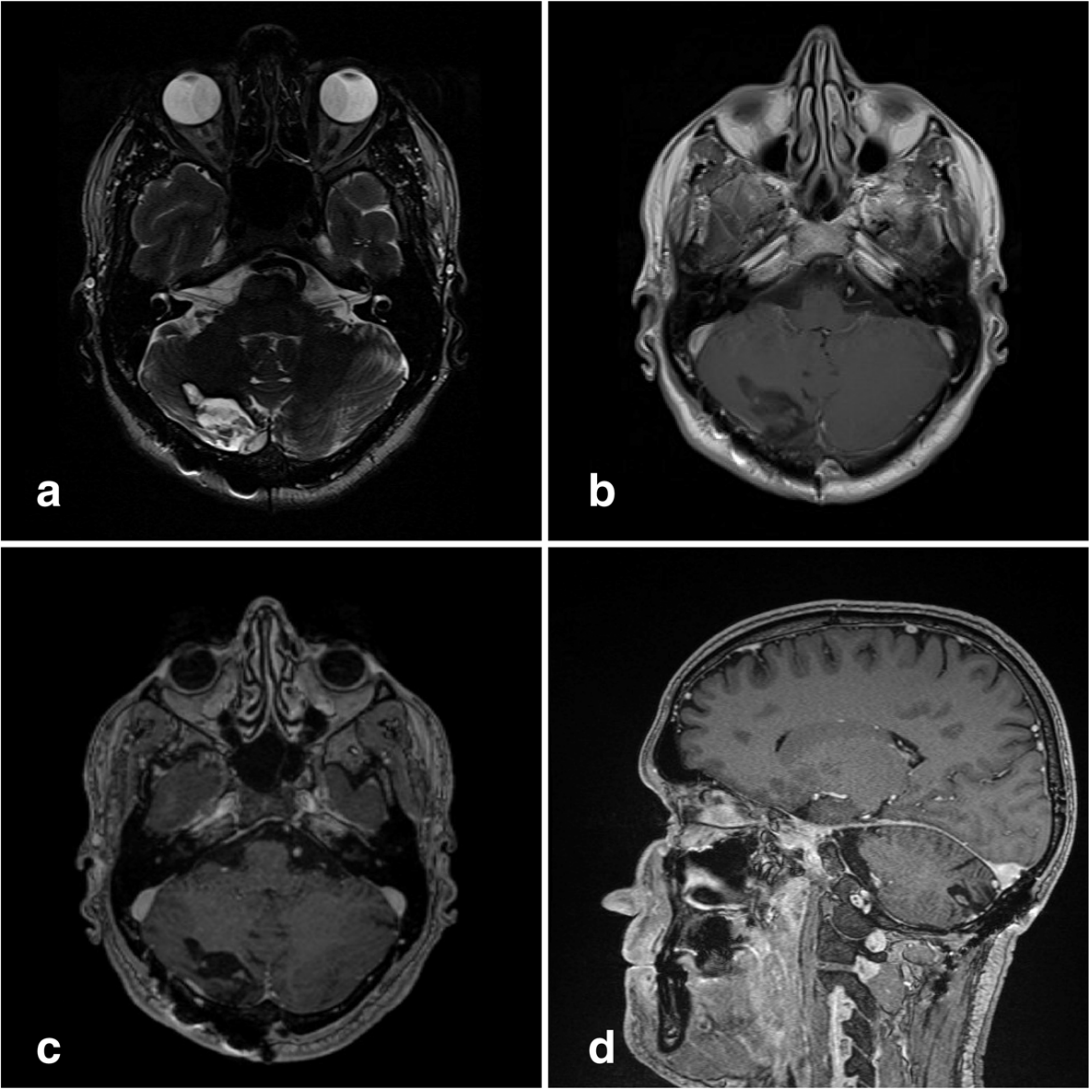
Postoperative magnetic resonance imaging 15 months after surgery shows no recurrence of the tumor on T2-weighted imaging (**a**) and on T1-weighted imaging (**b**). No contrast enhancement can be seen on T1-weighted images with contrast enhancement (**c** + **d**)

A postoperative CT scan revealed a regular finding without hydrocephalus and hemorrhage. Our patient was observed in our neurosurgical intensive care unit for one night and was transferred to a general ward the following day without neurological deficiency. Postoperative MRI, which was performed 48 hours after surgery, showed no residual tumor. His postoperative course was uneventful. He received no adjuvant treatment and there has been no evidence of tumor recurrence over a period of 15 months (Fig. 2). A neurological examination at last follow-up, 15 months after surgery, revealed no neurological deficits. The preoperative nausea and headache he experienced had stopped.

**Fig. 3:**
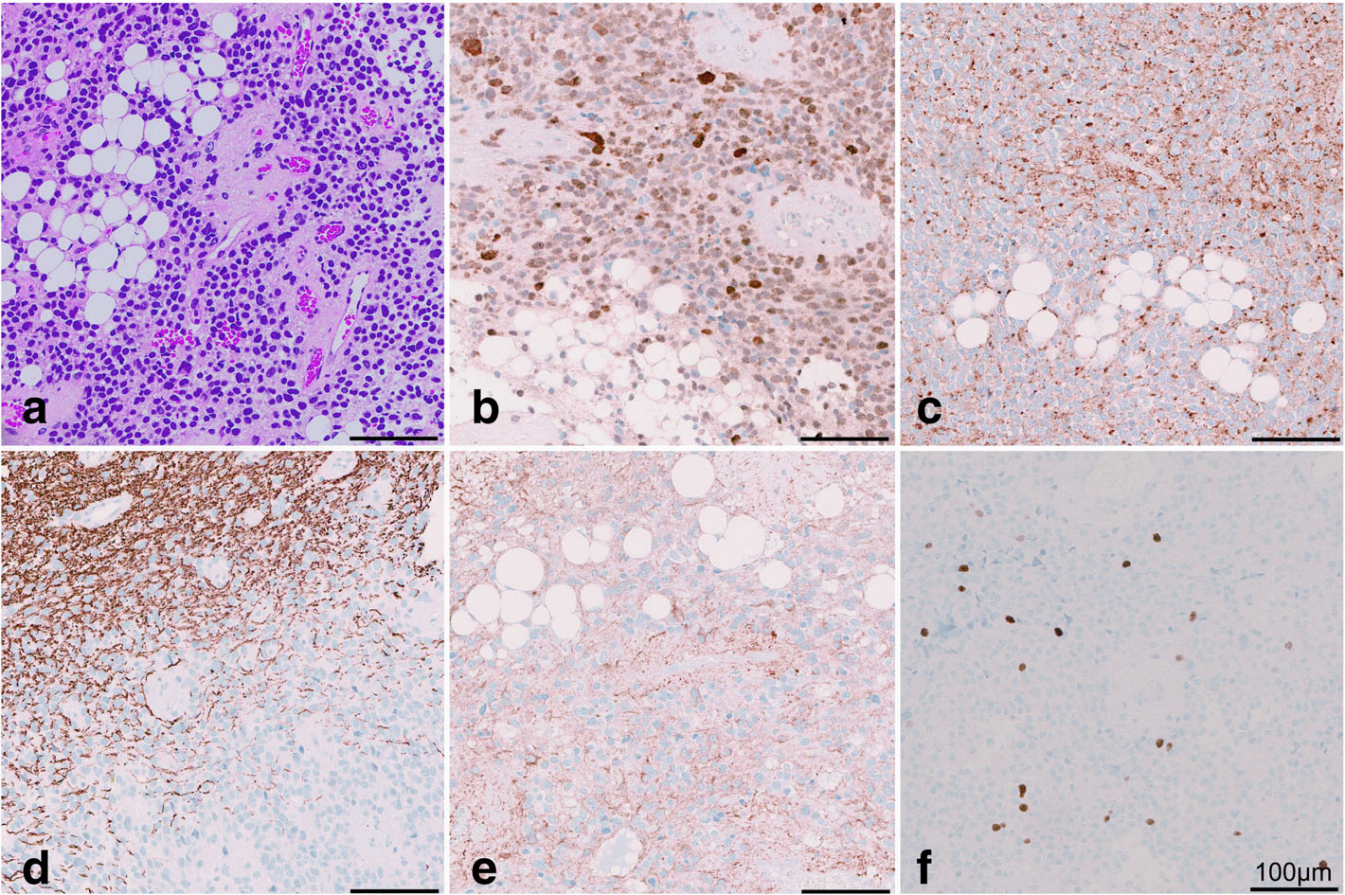
Liponeurocytoma with typical histology with focal accumulation of adipocytic tumor cells; hematoxylin and eosin (**a**). Small tumor cells and neoplastic cells with lipomatous differentiation express NeuN (**b**) and synaptophysin (**c**). Axons of the surrounding central nervous system tissue express neurofilament (**d**). Expression of the astrocytic marker glial fibrillary acid protein is observed focally (**e**). The Ki-67 proliferation index, as determined by nuclear MIB1 monoclonal antibody staining, is low; approximately 2% (**f**)

On histopathological examination, hematoxylin and eosin-stained paraffin sections showed predominantly small to moderately cellular tumor growing compactly, sometimes diffuse, infiltrating the surrounding cerebellar tissue. Tumor cells contained mainly light eosinophilic, sometimes clear, cytoplasm and round to oval nuclei and smaller nucleoli. Some tumor cells showed an astrocytic differentiation. Furthermore, around 10% of the tumor area comprised focal lipidized cells (Fig. 3a). No significant mitotic activity, < 1 mitosis/20 high-power field (HPF), and no necrosis were observed. In immunohistochemical analysis NeuN (Fig. 3b) was detected in 80% and synaptophysin (Fig. 3c) was detected in 30% of the non-lipomatous cells. Tumor cells were negative for neurofilament (Fig. 3d; surrounding CNS tissue stained positive) and chromogranin A. Glial fibrillary acid protein (GFAP) was observed in 20% of the tumor cells (Fig. 3e). Ki-67/MIB-1 proliferation index (Fig. 3f), as determined by nuclear MIB1 monoclonal antibody staining, was around 2% (Fig. 3).

## Discussion

This case report describes an unusual case of a 39-year-old Italian man suffering from a liponeurocytoma located in the posterior fossa. Complete tumor resection was achieved and postoperative MRI revealed no residual tumor. Follow-up 15 months after surgery showed no tumor recurrence on MRI; our patient was asymptomatic. A neurological examination at follow-up revealed no neurological deficits. The radiological diagnostics, histopathological findings, differential diagnoses, and the possible adjuvant therapy are described in detail. Therefore, this case report offers an informative overview about this uncommon tumor entity.

Liponeurocytoma is a rare, benign tumor most commonly located in the posterior fossa. Mean age at the time of diagnosis is around 40 to 50 years of age. Most of the patients are more than 30 years of age. No gender predominance has been reported according to the study by Patel *et al.* [4]. Typically, symptoms may include headache, vomiting, nausea, and dizziness as well as ataxia with unsteadiness and frequent falls [5]. A flow obstruction of the cerebrospinal fluid may also be present [6].

Liponeurocytoma is a neurocytic neoplasm with a variable extent of neuronal/neurocytic differentiation and a focal lipomatous and astrocytic differentiation reflected by a consistent immunoreactivity to synaptophysin, neuron-specific enolase (NSE), and MAP-2. The tumor cells can also be positive for nuclear NeuN. They are negative for cytoplasmic IDH1- R132H, epithelial membrane antigen (EMA), cytokeratin (CK), and nuclear Olig-2. It shows a monotonous pattern of isomorphic round to oval cells with focal accumulations of lipid-laden cells. The lipomatous cells of the liponeurocytoma seem to be neuroepithelial tumor cells with lipid accumulation or they constitute a form of lipidization instead of true adipose cells [7, 8]. In the lipomatous cells, the lipid vacuole membranes were positive for SYN, MAP-2, vimentin, and S-100. The expression of GFAP and S-100 is limited to scattered reactive astrocytes [9–11].

On radiological examination, liponeurocytomas often show a more or less well-demarcated hypointense mass with attenuation values of fatty tissue on CT scans. The tumor may be associated with parenchymal cysts or cerebellar hemorrhage [12]. MRI of a cerebellar liponeurocytoma normally shows an isointensity on T1-weighted imaging, a heterogeneous intensity on T2-weighted imaging, and a high intensity on diffusion-weighted imaging. No or in some cases a mild surrounding edema may be observed. On FLAIR imaging a slight hyperintensity and on diffusion-weighted imaging a high intensity may be seen. A light contrast enhancement can be seen after administration of contrast agent [13, 14].

Differential diagnoses include oligodendrogliomas, clear cell ependymomas, or high-grade tumors like medulloblastomas. Oligodendrogliomas may show similar pathological findings demonstrating neuronal differentiation with glial and neuronal characteristics. Oligodendrogliomas show highly positive results of Olig2 immunohistochemistry [15] in contrast to liponeurocytomas [16]. Despite this, a chromosome 1p/19q co-deletion is shown in 50–80% of oligodendrogliomas, while 1p/19q deletion is not observed in liponeurocytomas [17, 18]. In addition, oligodendrogliomas show a high frequency of *isocitrate dehydrogenase 1* (*IDH1 R132H*) mutation in addition to 1p/19q co-deletion [19].

Medulloblastoma with lipidized cells and lipomatous ependymomas can be differentiated using immunohistochemical panels. Negative reaction for EMA rules out the possibility of an ependymoma. Older age of presentation (43.66 ± 15.60 years) and extensive lipomatous change combined with a lower Ki-67/MIB-1 proliferation index favor a diagnosis of a liponeurocytoma.

Medulloblastomas show hyperdense areas with calcification in around 20% of cases on CT scans and a homogenous contrast enhancement can be observed on CT scans with contrast enhancement. On MRI, a hypointense signal on T1-weighted images and an isointense to hyperintense signal on T2-weighted images can be observed for medulloblastomas. A hyperintense FLAIR image can be detected. Diffusion-weighted images of medulloblastomas are hyperintense and on T1-weighted images with contrast enhancement medulloblastomas normally present a heterogeneous strong contrast enhancement [20].

Furthermore, Horstmann *et al.* analyzed the genetic profile of a liponeurocytoma [20]. They were able to show that liponeurocytoma is a distinct tumor entity that is genetically different from medulloblastomas. There was no case with *PTCH*, *APC*, or *β-catenin* mutations, known to be present in subsets of medulloblastomas. Furthermore, they suggested a relationship to central neurocytomas caused by the complementary DNA (cDNA) expression array data, but the presence of *TP53* mutations, which are absent in central neurocytomas, suggests that their genetic pathways are different [21].

Fluorescent *in situ* hybridization studies of a liponeurocytoma showed no evidence of 1p/19q co-deletion, *N-MYC* or epidermal growth factor receptor amplification, *MYC* or EWSR1 rearrangements, or PTEN deletion [22, 23]. In contrast to these findings, large cell/anaplastic medulloblastomas are associated with *C-MYC* or *N-MYC* amplifications [23]. Despite this, Wolf *et al.* [24] and Pikis *et al*. [16] suggested an inheritable predisposition for tumor development.

The absence of mitoses, necrosis, vascular hyperplasia, and nucleo-cytoplasmic atypia, and the low proliferation rate (MIB-1 < 6%) favor the interpretation of a benign lesion. But, Cacciola *et al.* showed in their review of the literature a recurrence in 20 to 32% of patients at a mean time of 10 years after surgery [25]. Interestingly, patients treated by surgery alone showed a recurrence rate of 50% [25]. Châtillon *et al.* reported a recurrence rate of 50% in patients treated by subtotal resection without adjuvant radiotherapy [26]. These findings are in line with those from Limaiem *et al*., who were able to show mean time until recurrence of around 8.5 years [27]. In addition, they suggested that the tumor may recur due to mitoses present in the lesion and > 10% Ki-67 positive cells [27]. Radke *et al*. were able to show an increase of Ki-67 positive cells in recurrent tumors [23]. Due to the potentially long-term malignancy, close follow-up is recommended over that period [27, 28].

Surgery is the primary treatment of choice, but the following postoperative treatment strategy is still controversial and under discussion due to the benign character of the tumor. Long-term follow-up examinations showed a tumor recurrence in 20 to 32% of the patients at a mean time of 8 to 10 years after surgery. In addition, recurrent tumor growth is more common in patients where total resection was not achieved. Therefore, additional radiotherapy has to be discussed in those patients [25, 27]. Jackson *et al.* showed in their review of the literature that a recurrence was not observed in those patients who received adjuvant radiotherapy [29]. In contrast to this, 50% of the patients who did not receive an adjuvant therapy suffered from a tumor recurrence [29]. Therefore, adjuvant therapy seems to have a favorable impact on tumor recurrence.

In summary, the present study reported a case of cerebellar liponeurocytoma that was successfully treated by total resection without tumor recurrence 15 months after surgery.

## Conclusions

Liponeurocytomas are rare benign tumors occurring mainly in the cerebellum. They show a different genetic pathway compared to central neurocytomas and medulloblastomas. Surgery is the primary therapy of choice. Adjuvant radiotherapy seems to be sufficient to avoid a tumor recurrence.

## Abbreviations

CNS: Central nervous system
CT: Computed tomography
EMA: Epithelial membrane antigen
FLAIR: Fluid-attenuated inversion recovery
GFAP: Glial fibrillary acid protein
MRI: Magnetic resonance imaging
WHO: World Health Organization

## References

[1] Bechtel JT, Patton JM, Takei Y. Mixed mesenchymal and neuroectodermal tumor of the cerebellum. Acta Neuropathol. 1978;41:261–3. 10.1007/BF00690447 206094

[2] Radner H, Blumcke I, Reifenberger G, Wiestler OD. The new WHO classification of tumors of the nervous system 2000. Pathology and genetics. Pathologe. 2002;23:260–83. 10.1007/s00292-002-0530-8 12185780

[3] Louis DN, Perry A, Reifenberger G, von Deimling A, Figarella-Branger D, Cavenee WK, Ohgaki H, Wiestler OD, Kleihues P, Ellison DW. The 2016 World Health Organization Classification of Tumors of the Central Nervous System: a summary. Acta Neuropathol. 2016;131:803–20. 10.1007/s00401-016-1545-1 27157931

[4] Patel N, Fallah A, Provias J, Jha NK. Cerebellar liponeurocytoma. Can J Surg. 2009;52:E117–9. PMC2724827 19680499

[5] Chakraborti S, Mahadevan A, Govindan A, Yasha TC, Santosh V, Kovoor JM, Ramamurthi R, Alapatt JP, Hedge T, Shankar SK. Supratentorial and cerebellar liponeurocytomas: report of four cases with review of literature. J Neuro-Oncol. 2011;103:121–7. 10.1007/s11060-010-0361-z 20803304

[6] Gupta K, Salunke P, Kalra I, Vasishta RK. Central liponeurocytoma: case report and review of literature. Clin Neuropathol. 2011;30:80–5. 10.5414/npp30080 21329617

[7] Xu L, Du J, Wang J, Fang J, Liu Z, He Y, Li G. The clinicopathological features of liponeurocytoma. Brain Tumor Pathol. 2017;34:28–35. 10.1007/s10014-017-0279-7 28236115

[8] Louis DN, Ohgaki H, Wiestler OD, Cavenee WK, Burger PC, Jouvet A, Scheithauer BW, Kleihues P. The 2007 WHO classification of tumours of the central nervous system. Acta Neuropathol. 2007;114:97–109. 10.1007/s00401-007-0243-4 PMC1929165 17618441

[9] Rajesh L, Jindal A, Banjerjee A. Liponeurocytoma of lateral ventricle. Neurol India. 2010;58:805–6. 10.4103/0028-3886.72195 21045527

[10] Jouvet A, Lellouch-Tubiana A, Boddaert N, Zerah M, Champier J, Fèvre-Montange M. Fourth ventricle neurocytoma with lipomatous and ependymal differentiation: a case report. Acta Neuropathol. 2005;109:346–51. 10.1007/s00401-004-0963-7 15627205

[11] George D, Scheithauer B. Central Liponeurocytoma: Case Report. A J Surg Pathol. 2001;25:1551–5. 10.1097/00000478-200112000-00013 11717547

[12] Kuchelmeister K, Nestler U, Siekmann R, Schachenmayr W. Liponeurocytoma of the left lateral ventricle--case report and review of the literature. Clin Neuropathol. 2006;25:86–94. 16550742

[13] Takami H, Mukasa A, Ikemura M, Shibahara J, Takahashi M, Momose T, Saito N. Findings from positron emission tomography and genetic analyses for cerebellar liponeurocytoma. Brain Tumor Pathol. 2015;32:210–5. 10.1007/s10014-014-0210-4 25527206

[14] Beizig N, Ziadi S, Ladib M, Mokni M. Cerebellar liponeurocytoma: case report. Neurochirurgie. 2013;59:39–42. 10.1016/j.neuchi.2012.09.002 23158671

[15] Marie Y, Sanson M, Mokhtari K, Leuraud P, Kujas M, Delattre JY, Poirier J, Zalc B, Hoang-Xuan K. OLIG2 as a specific marker of oligodendroglial tumour cells. Lancet. 2001;358:298–300. 10.1016/S0140-6736(01)05499-X 11498220

[16] Pikis S, Fellig Y, Margolin E. Cerebellar liponeurocytoma in two siblings suggests a possible familial predisposition. J Clin Neurosci. 2016;32:154–6. 10.1016/j.jocn.2016.04.004 27349466

[17] Reifenberger G, Louis DN. Oligodendroglioma: toward molecular definitions in diagnostic neuro-oncology. J Neuropathol Exp Neurol. 2003;62:111–26. 10.1093/jnen/62.2.111 12578221

[18] Weller M, Stupp R, Hegi ME, van den Bent M, Tonn JC, Sanson M, Wick W, Reifenberger G. Personalized care in neurooncology coming of age: why we need MGMT and 1p/19q testing for malignant glioma patients in clinical practice. Neuro-Oncology. 2012;14(Suppl 4):iv100–8. 10.1093/neuonc/nos206 PMC3480248 23095825

[19] Labussiere MIA, Wang X-W, Marie Y, Boisselier B, Falet C, Paris S, Laffaire J, Carpentier C, Criniere E. All the 1p19q codeleted gliomas are mutated on *IDH1* or *IDH2*. Neurology. 2010;74:1886–90. 10.1212/WNL.0b013e3181e1cf3a 20427748

[20] Koeller KK, Rushing EJ. From the archives of the AFIP: medulloblastoma: a comprehensive review with radiologic-pathologic correlation. Radiographics. 2003;23:1613–37. 10.1148/rg.236035168 14615567

[21] Horstmann S, Perry A, Reifenberger G, Giangaspero F, Huang H, Hara A, Masuoka J, Rainov NG, Bergmann M, Heppner FL, et al. Genetic and expression profiles of cerebellar liponeurocytomas. Brain Pathol. 2004;14:281–9. 10.1111/j.1750-3639.2004.tb00065.x PMC8095792 15446583

[22] Tucker A, Boon-Unge K, McLaughlin N, Ibrahim H, Rao N, Martin N, Everson R, Khanlou N. Cerebellar Liponeurocytoma: Relevant Clinical Cytogenetic Findings. J Pathol Transl Med. 2017;51:335–40. 10.4132/jptm.2016.07.24 PMC5445196 27750407

[23] Radke J, Gehlhaar C, Lenze D, Capper D, Bock A, Heppner FL, Jodicke A, Koch A. The evolution of the anaplastic cerebellar liponeurocytoma: case report and review of the literature. Clin Neuropathol. 2015;34:19–25. 10.5414/NP300783 25250652

[24] Wolf A, Alghefari H, Krivosheya D, Staudt MD, Bowden G, Macdonald DR, Goobie S, Ramsay D, Hebb MO. Cerebellar liponeurocytoma: a rare intracranial tumor with possible familial predisposition. Case report. J Neurosurg. 2016;125:57–61. 10.3171/2015.6.JNS142965 26613167

[25] Cacciola F, Conti R, Taddei GL, Buccoliero AM, Di Lorenzo N. Cerebellar liponeurocytoma. Case report with considerations on prognosis and management. Acta Neurochir. 2002;144:829–33. 10.1007/s007010200082 12181694

[26] Châtillon CE, Guiot MC, Roberge D, Leblanc R. Cerebellar liponeurocytoma with high proliferation index: treatment options. Can J Neurol Sci. 2009;36:658–61. 10.1017/s0317167100008210 19831140

[27] Limaiem F, Bellil S, Chelly I, Bellil K, Mekni A, Jemel H, Haouet S, Zitouna M, Kchir N. Recurrent cerebellar liponeurocytoma with supratentorial extension. Can J Neurol Sci. 2009;36:662–5. 10.1017/s0317167100008222 19831141

[28] Aker F, Ozkara S, Eren P, Peker O, Armağan S, Hakan T. Cerebellar liponeurocytoma/lipodized medulloblastoma. J Neuro-Oncol. 2005;71:53–9. 10.1007/s11060-004-9172-4 15719276

[29] Jackson TR, Regine WF, Wilson D, Davis DG. Cerebellar liponeurocytoma. Case report and review of the literature. J Neurosurg. 2001;95:700–3. 10.3171/jns.2001.95.4.0700 11596966

